# Characterization of micro-RNA in women with different ovarian reserve

**DOI:** 10.1038/s41598-021-92901-w

**Published:** 2021-06-25

**Authors:** Masood Abu-Halima, Lea Simone Becker, Basim M. Ayesh, Simona Lucia Baus, Amer Hamza, Ulrike Fischer, Mohamad Hammadeh, Andreas Keller, Eckart Meese

**Affiliations:** 1 Institute of Human Genetics, Saarland University, 66421 Homburg, Saar Germany; 2 Department of Laboratory Medical Sciences, Alaqsa University, Gaza, Palestine; 3 Department of Obstetrics and Gynecology, Saarland University, 66421 Homburg, Saar Germany; 4 Kantonspital Baden, Im Ergel 1, 5400 Baden, Switzerland; 5 Chair for Clinical Bioinformatics, Saarland University, 66123 Saarbruecken, Germany

**Keywords:** Genetics, Molecular biology, Molecular medicine

## Abstract

Women undergoing infertility treatment are routinely subjected to one or more tests of ovarian reserve. Therefore, an adequate assessment of the ovarian reserve is necessary for the treatment. In this study, we aimed to characterize the potential role of microRNAs (miRNAs) as biomarkers for women with different ovarian reserves. A total of 159 women were recruited in the study and classified according to their anti-Müllerian hormone (AMH) level into three groups: (1) low ovarian reserve (LAMH, n = 39), (2) normal ovarian reserve (NAMH, n = 80), and (3) high ovarian reserve (HAMH, n = 40). SurePrint Human miRNA array screening and reverse transcription-quantitative PCR (RT-qPCR) were respectively employed to screen and validate the miRNA abundance level in the three tested groups. Compared with NAMH, the abundance level of 34 and 98 miRNAs was found to be significantly altered in LAMH and HAMH, respectively. The abundance level of miRNAs was further validated by RT-qPCR in both, the screening samples as well as in an independent set of validation samples. The abundance levels of the validated miRNAs were significantly correlated with the AMH level. The best AUC value for the prediction of the increase and decrease in the AMH level was obtained for the miR-100-5p and miR-21-5p, respectively. The level of miRNAs abundance correlates with the level of AMH, which may serve as a tool for identifying women with a different ovarian reserve and may help to lay the ground for the development of novel diagnostic approaches.

## Introduction

Assessment of the ovarian reserve has become essential to determine the strategy for the treatment of female infertility. For this purpose, several non-invasive clinical, endocrinological, and ultrasonographic examinations, at the early follicular phase, have been applied. Yet, to our best knowledge, no ideal test for ovarian reserve assessment exists. Serum levels of anti-Müllerian hormone (AMH) and total antral follicle count (AFC) performed better than all other known markers, and are the most predictive, direct tests for evaluation of ovarian reserve^1^. Currently, no molecular biomarkers are used, in combination with these conventional tests, to brush up the predictive accuracy of different forms of ovarian reserves. MicroRNAs (miRNAs) have received increasing attention due to their likely role in the regulation of nearly every cellular process. Currently, there are 2300 “real” miRNAs in the miRNA database based on miRBase.org^2^. Changes in miRNA abundance level are associated with many reproductive pathologies in both male and female partners^3–11^. In the female partner, miRNAs were found to be involved in ovarian development and function^11^. Specifically, the literature supports a crucial role for miRNAs during various stages in either somatic cells and/or ovarian follicles. In turn, a functional and crucial role in the production of the mature, and viable oocytes that are capable of fertilization and subsequent embryo development and implantation of miRNA has been suggested^12–14^. Circulating miRNAs have been utilized as a potential independent predictive system for different diseases due to their abundant and unique merits in body fluid (i.e., stable, easy to be detected, and potentially disease-specific)^15,16^. As miRNAs seem to be an important regulator of gene expression during follicular development and maturation, we hypothesized that circulating miRNAs in the early follicular phase could serve as potentially useful biomarkers for predicting ovarian reserve. To address this hypothesis, we characterized the abundance level of circulating miRNAs in women undergoing assisted reproductive technology (ART) using a large panel of human miRNA arrays. Then, we validated the deregulated miRNAs in a large independent cohort of samples with individual quantitative reverse transcriptase-polymerase chain reaction (RT-qPCR) assays. Data were analyzed to determine whether circulating miRNA profiles correlate with the level of serum AMH and can serve as potential biomarkers for predicting ovarian reserve in women attending infertility treatment.

## Results

### Study population

The demographic, hormonal, and clinical characteristics of the participating women presenting at an infertility clinic were collected and statistically evaluated. The women undergoing infertility treatment were classified according to the ovarian reserve into three groups; normal (n = 80), high (n = 40), and low (n = 39) based on AMH level as described by Antonio La Marca and Sunkara^1^. As shown in Table 1, no statistically significant difference was observed between the groups in terms of their mean FT4 and TSH. However, a statistically significant difference was detected between the three groups in terms of the mean age, PRL, LH, FSH, Basal E2, Testosterone, Androstenedione, DHEA-S, and AFC (*P* < 0.05).

**Table 1:** Statistical evaluation of the demographic, hormonal and clinical characteristics of women presenting at an infertility clinic.

Variable	Normal response (n = 80)	High response (n = 40)	Low response (n = 39)	*P *value
Age (year)	32 ± 0.50	30 ± 0.65	36 ± 0.61	0.0001
FT4 (ng/dL)	1.8 ± 0.30	1.4 ± 0.19	1.6 ± 0.36	0.6892
TSH (μIU/mL)	2.0 ± 0.17	2.2 ± 0.28	2.1 ± 0.20	0.7342
PRL (µIU/mL)	346 ± 17.0	311 ± 19	271 ± 16	0.0185
LH (mIU/mL)	6.2 ± 0.34	7.9 ± 0.68	8.1 ± 0.84	0.0221
FSH (mIU/mL)	7.1 ± 0.20	6.3 ± 0.23	14.0 ± 1.7	0.0001
Basal E2 (pg/mL)	40 ± 2.40	37 ± 3.00	54 ± 4.9	0.0012
Total Testosterone (ng/mL)	0.25 ± 0.017	0.38 ± 0.024	0.19 ± 0.015	0.0001
Androstenedione (ng/mL)	1.7 ± 0.09	2.4 ± 0.18	1.2 ± 0.09	0.0001
DHEA-S (ng/mL)	1705 ± 101	1905 ± 154	1388 ± 113	0.0304
AMH (ng/mL)	2.6 ± 0.11	7.3 ± 0.33	0.51 ± 0.05	0.0001
AFC	11 ± 0.71	20 ± 1.5	5.0 ± 0.43	0.0001

n = 159. Grouping according to normal, high, and low ovarian reserve.

ANOVA, mean ± standard deviation.

Statistically significant if P value < 0.05.

*FT4* free thyroxine 4, *TSH* thyroid stimulating hormone, *PRL* prolactin, *LH* luteinizing hormone, *FSH* follicle stimulating hormone, *Basal E2* basal estradiol, *DHEA-S* dehydroepiandrosterone sulfate, *AMH* anti-Müllerian hormone, *AFC* antral follicle count.

To determine which of the demographic, hormonal and clinical characteristics correlated directly with the AMH level, a spearman's correlation was carried out. The serum level of AMH was negatively correlated with age (*P* = 0.001; r = − 0.49) and serum FSH concentration (*P* = 0.003; r = 0.45) and positively correlated with the AFC (*P* < 0.0001; r = 0.694). In addition, weak positive correlations were found between the serum concentrations of AMH and LH (*P* < 0.033; r = 0.34), Testosterone (*P* = 0.018; r = 0.37), and Androstenedione (*P* = 0.048; r = 0.31). No significant correlation was found between the serum concentration of AMH and basal E2, DHEA-S, PRL, and FT4 (Supplementary Table 1).

### Screening of differentially abundant miRNAs using microarray

**Figure 1:**
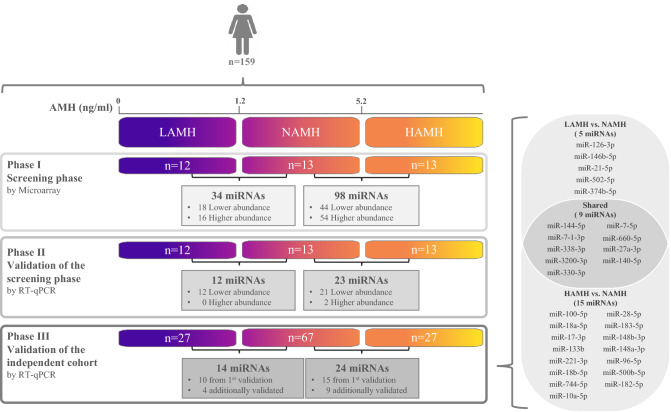
Workflow chart showing the number of women enrolled and the number and regulation of the detected miRNAs by microarray and RT-qPCR analyses.

To identify miRNAs that are differentially abundant in the blood samples of included women, we analyzed the abundance level of 2549 human mature miRNAs of miRBase v21. The abundance levels of circulating miRNAs were screened in 38 women including LAMH (n = 12), NAMH (n = 13), and HAMH (n = 13) (phase I). By considering the miRNAs with a significant adjusted *P *value of < 0.05 with fold change ≥ 2 (lower and higher abundant level), only 34 and 98 miRNAs showed differential abundance levels in LAMH and HAMH groups, as compared to NAMH group, respectively (Fig. 1). As shown in Table 2, of the 34 differentially abundant miRNAs in LAMH versus NAMH, 18 miRNAs were significantly lower and 16 miRNAs were significantly higher in the abundance level, whereas 44 miRNAs were significantly lower, and 54 miRNAs were significantly higher in the HAMH versus NAMH group. The two abnormal groups i.e., LAMH versus HAMH were also compared with one another, however, no significantly differently abundant miRNA was identified (data not shown).

**Table 2:** Significantly abundant miRNAs in the blood of women presenting at an infertility clinic as determined by microarray (adjusted *P* < 0.05 and fold change ≥ 2).

miRBase Accession Nr	MicroRNA	Adjusted *P* value	P value	Log2 FC	Regulation
**LAMH (n = 12) compared to NAMH group (n = 13)**					
MIMAT0004600	miR-144-5p	0.0309	0.0038	− 3.69	Lower
MIMAT0004955	miR-374b-5p	0.0370	0.0063	− 3.38	Lower
MIMAT0004553	miR-7-1-3p	0.0259	0.0005	− 3.33	Lower
MIMAT0000431	miR-140-5p	0.0413	0.0087	− 3.02	Lower
MIMAT0000445	miR-126-3p	0.0475	0.0134	− 2.96	Lower
MIMAT0002809	miR-146b-5p	0.0273	0.0010	− 2.77	Lower
MIMAT0000763	miR-338-3p	0.0308	0.0027	− 2.74	Lower
MIMAT0015085	miR-3200-3p	0.0390	0.0069	− 2.62	Lower
MIMAT0005796	miR-1271-5p	0.0390	0.0078	− 2.19	Lower
MIMAT0004586	miR-15b-3p	0.0475	0.0127	− 2.10	Lower
MIMAT0002873	miR-502-5p	0.0274	0.0018	− 2.10	Lower
MIMAT0000084	miR-27a-3p	0.0309	0.0032	− 2.10	Lower
MIMAT0000263	miR-199b-5p	0.0475	0.0126	− 1.48	Lower
MIMAT0000751	miR-330-3p	0.0494	0.0145	− 1.29	Lower
MIMAT0000759	miR-148b-3p	0.0309	0.0038	− 1.17	Lower
MIMAT0000076	miR-21-5p	0.0278	0.0020	− 1.17	Lower
MIMAT0000252	miR-7-5p	0.0273	0.0012	− 1.16	Lower
MIMAT0003338	miR-660-5p	0.0273	0.0016	− 1.10	Lower
MIMAT0022694	miR-211-3p	0.0118	0.0001	4.38	Higher
MIMAT0019835	miR-4721	0.0007	0.0000	4.29	Higher
MIMAT0005942	miR-1288-3p	0.0273	0.0014	3.38	Higher
MIMAT0027460	miR-6780a-5p	0.0273	0.0013	3.31	Higher
MIMAT0027514	miR-6807-5p	0.0308	0.0027	3.27	Higher
MIMAT0019771	miR-4685-5p	0.0308	0.0024	3.11	Higher
MIMAT0025480	miR-6512-5p	0.0308	0.0030	3.08	Higher
MIMAT0003240	miR-575	0.0318	0.0041	2.93	Higher
MIMAT0014990	miR-3127-5p	0.0475	0.0127	2.82	Higher
MIMAT0025855	miR-6723-5p	0.0475	0.0120	2.72	Higher
MIMAT0015030	miR-3156-5p	0.0309	0.0038	2.58	Higher
MIMAT0021125	miR-5194	0.0390	0.0074	2.36	Higher
MIMAT0024599	miR-6126	0.0475	0.0126	2.35	Higher
MIMAT0004982	miR-939-5p	0.0425	0.0099	2.14	Higher
MIMAT0022275	miR-5581-5p	0.0475	0.0135	2.10	Higher
MIMAT0027434	miR-6767-5p	0.0475	0.0137	2.08	Higher
**HAMH (n = 13) compared to NAMH group (n = 13)**					
MIMAT0000098	miR-100-5p	0.0453	0.0309	− 3.73	Lower
MIMAT0015085	miR-3200-3p	0.0262	0.0010	− 3.00	Lower
MIMAT0022482	miR-5690	0.0262	0.0021	− 2.95	Lower
MIMAT0004553	miR-7-1-3p	0.0263	0.0044	− 2.87	Lower
MIMAT0000095	miR-96-5p	0.0437	0.0165	− 2.67	Lower
MIMAT0016925	miR-500b-5p	0.0282	0.0054	− 2.67	Lower
MIMAT0004600	miR-144-5p	0.0453	0.0312	− 2.67	Lower
MIMAT0003258	miR-590-5p	0.0453	0.0226	− 2.65	Lower
MIMAT0000431	miR-140-5p	0.0453	0.0178	− 2.60	Lower
MIMAT0000763	miR-338-3p	0.0363	0.0089	− 2.48	Lower
MIMAT0000259	miR-182-5p	0.0363	0.0089	− 2.34	Lower
MIMAT0002873	miR-502-5p	0.0262	0.0023	− 2.32	Lower
MIMAT0004560	miR-183-3p	0.0262	0.0034	− 2.29	Lower
MIMAT0000072	miR-18a-5p	0.0453	0.0284	− 2.28	Lower
MIMAT0000071	miR-17-3p	0.0453	0.0360	− 2.24	Lower
MIMAT0000770	miR-133b	0.0453	0.0323	− 2.23	Lower
MIMAT0004814	miR-654-3p	0.0453	0.0385	− 2.13	Lower
MIMAT0004586	miR-15b-3p	0.0453	0.0196	− 2.11	Lower
MIMAT0000278	miR-221-3p	0.0474	0.0490	− 2.06	Lower
MIMAT0001412	miR-18b-5p	0.0453	0.0391	− 2.03	Lower
MIMAT0000689	miR-99b-5p	0.0453	0.0315	− 1.99	Lower
MIMAT0004945	miR-744-5p	0.0453	0.0277	− 1.98	Lower
MIMAT0000084	miR-27a-3p	0.0363	0.0090	− 1.96	Lower
MIMAT0005593	miR-1238-3p	0.0453	0.0314	− 1.89	Lower
MIMAT0000257	miR-181b-5p	0.0462	0.0412	− 1.84	Lower
MIMAT0000426	miR-132-3p	0.0387	0.0121	− 1.83	Lower
MIMAT0000253	miR-10a-5p	0.0453	0.0356	− 1.81	Lower
MIMAT0000085	miR-28-5p	0.0453	0.0180	− 1.67	Lower
MIMAT0004558	miR-181a-2-3p	0.0453	0.0389	− 1.60	Lower
MIMAT0017392	miR-3200-5p	0.0282	0.0054	− 1.49	Lower
MIMAT0000243	miR-148a-3p	0.0453	0.0197	− 1.41	Lower
MIMAT0004810	miR-629-5p	0.0453	0.0378	− 1.39	Lower
MIMAT0000252	miR-7-5p	0.0262	0.0008	− 1.31	Lower
MIMAT0000751	miR-330-3p	0.0370	0.0111	− 1.29	Lower
MIMAT0000279	miR-222-3p	0.0453	0.0215	− 1.27	Lower
MIMAT0003338	miR-660-5p	0.0394	0.0131	− 1.26	Lower
MIMAT0000452	miR-154-5p	0.0453	0.0388	− 1.25	Lower
MIMAT0000261	miR-183-5p	0.0262	0.0035	− 1.21	Lower
MIMAT0000263	miR-199b-5p	0.0480	0.0502	− 1.19	Lower
MIMAT0002876	miR-505-3p	0.0465	0.0477	− 1.18	Lower
MIMAT0019923	miR-4769-3p	0.0495	0.0528	− 1.09	Lower
MIMAT0000759	miR-148b-3p	0.0372	0.0114	− 1.09	Lower
MIMAT0000231	miR-199a-5p	0.0262	0.0029	− 1.05	Lower
MIMAT0000418	miR-23b-3p	0.0462	0.0454	− 1.04	Lower
MIMAT0019835	miR-4721	0.0262	0.0009	3.91	Higher
MIMAT0022694	miR-211-3p	0.0282	0.0052	3.29	Higher
MIMAT0005942	miR-1288-3p	0.0262	0.0032	3.17	Higher
MIMAT0027460	miR-6780a-5p	0.0282	0.0056	2.96	Higher
MIMAT0027470	miR-6785-5p	0.0462	0.0442	2.67	Higher
MIMAT0027514	miR-6807-5p	0.0393	0.0128	2.64	Higher
MIMAT0022942	miR-1229-5p	0.0453	0.0353	2.59	Higher
MIMAT0022271	miR-664b-5p	0.0453	0.0368	2.50	Higher
MIMAT0019849	miR-4728-5p	0.0370	0.0109	2.49	Higher
MIMAT0002877	miR-513a-5p	0.0453	0.0265	2.49	Higher
MIMAT0024599	miR-6126	0.0437	0.0160	2.42	Higher
MIMAT0019871	miR-4741	0.0453	0.0308	2.38	Higher
MIMAT0003240	miR-575	0.0453	0.0312	2.30	Higher
MIMAT0025480	miR-6512-5p	0.0453	0.0301	2.28	Higher
MIMAT0005948	miR-664a-5p	0.0453	0.0283	2.27	Higher
MIMAT0014988	miR-3125	0.0462	0.0434	2.25	Higher
MIMAT0022286	miR-5585-3p	0.0363	0.0102	2.24	Higher
MIMAT0025855	miR-6723-5p	0.0453	0.0357	2.24	Higher
MIMAT0014990	miR-3127-5p	0.0453	0.0368	2.22	Higher
MIMAT0015030	miR-3156-5p	0.0453	0.0263	2.03	Higher
MIMAT0025476	miR-6510-5p	0.0453	0.0256	2.02	Higher
MIMAT0021033	miR-5006-5p	0.0453	0.0261	1.98	Higher
MIMAT0021125	miR-5194	0.0465	0.0477	1.91	Higher
MIMAT0018072	miR-3652	0.0323	0.0071	1.89	Higher
MIMAT0027355	miR-6727-5p	0.0475	0.0494	1.85	Higher
MIMAT0004761	miR-483-5p	0.0453	0.0263	1.81	Higher
MIMAT0019749	miR-4669	0.0453	0.0340	1.80	Higher
MIMAT0027524	miR-6812-5p	0.0453	0.0278	1.78	Higher
MIMAT0019035	miR-4499	0.0453	0.0256	1.76	Higher
MIMAT0027566	miR-6833-5p	0.0453	0.0317	1.74	Higher
MIMAT0016852	miR-4298	0.0453	0.0261	1.73	Higher
MIMAT0031016	miR-8089	0.0453	0.0264	1.70	Higher
MIMAT0018943	miR-4428	0.0462	0.0441	1.66	Higher
MIMAT0004918	miR-892b	0.0462	0.0424	1.65	Higher
MIMAT0019791	miR-4697-5p	0.0453	0.0311	1.61	Higher
MIMAT0022945	miR-1236-5p	0.0453	0.0293	1.46	Higher
MIMAT0027450	miR-6775-5p	0.0453	0.0333	1.45	Higher
MIMAT0019691	miR-4634	0.0453	0.0364	1.45	Higher
MIMAT0030999	miR-8072	0.0437	0.0162	1.44	Higher
MIMAT0027638	miR-6869-5p	0.0453	0.0338	1.38	Higher
MIMAT0018981	miR-4459	0.0453	0.0266	1.35	Higher
MIMAT0023701	miR-6076	0.0453	0.0320	1.35	Higher
MIMAT0005929	miR-1275	0.0363	0.0091	1.33	Higher
MIMAT0002816	miR-494-3p	0.0370	0.0108	1.33	Higher
MIMAT0019965	miR-4793-5p	0.0453	0.0363	1.30	Higher
MIMAT0027650	miR-6875-5p	0.0262	0.0009	1.30	Higher
MIMAT0027572	miR-6780b-5p	0.0262	0.0006	1.29	Higher
MIMAT0032116	miR-4485-5p	0.0262	0.0032	1.22	Higher
MIMAT0005880	miR-1290	0.0495	0.0528	1.21	Higher
MIMAT0029782	miR-7641	0.0262	0.0017	1.20	Higher
MIMAT0009448	miR-1973	0.0462	0.0444	1.09	Higher
MIMAT0027398	miR-6749-5p	0.0262	0.0013	1.05	Higher
MIMAT0015079	miR-3195	0.0263	0.0042	1.04	Higher
MIMAT0024597	miR-6124	0.0262	0.0015	1.01	Higher

LAMH (n = 12) compared to NAMH group (n = 13) and HAMH (n = 13) compared to NAMH group (n = 13).

Unpaired t test was used to calculate the *P *value.

Benjamini–Hochberg correction for multiple testing was used to adjust the *P *value.

Significant changes in abundance levels are shown with an adjusted *P *value < 0.05 and log2 fold change.

*LAMH* low anti-Müllerian hormone levels, *HAMH* high anti-Müllerian hormone levels, *NAMH* normal anti-Müllerian hormone level.

### Validation of candidate miRNAs by RT-qPCR

Using RT-qPCR, the validation of microarray data was performed to re-examine the abundance level of 74 miRNAs using the same samples, which have been used for the microarray experiments (phase II). These 74 miRNAs (as illustrated in Supplementary Fig. 1) were selected based on their differential abundance level in only LAMH (n = 7 miRNAs), only in HAMH (n = 46 miRNAs), and in both groups i.e., LAMH and HAMH (n = 21 miRNAs). As shown in Table 3 (phase II), RT-qPCR showed the same direction of abundance changes as the microarray analysis for 12 miRNAs, when comparing the samples from LAMH to NAMH (miR-144-5p, miR-7-1-3p, miR-126-3p, miR-146b-5p, miR-338-3p, miR-3200-3p, miR-330-3p, miR-21-5p, miR-7-5p, miR-660-5p, miR-148b-3p, and miR-199b-5p). Significant changes in abundance were confirmed for these 12 down-regulated miRNAs (*P* < 0.05).

**Table 3:** Validation of the abundant miRNAs in the blood of women presenting at an infertility clinic as determined by RT-qPCR (P < 0.05). LAMH (n = 12) compared to NAMH group (n = 13), HAMH (n = 13) compared to NAMH group (n = 13), LAMH (n = 27) compared to NAMH group (n = 67), and HAMH (n = 27) compared to NAMH group (n = 67).

LAMH (n = 12) compared to NAMH group (n = 13)				LAMH (n = 27) compared to NAMH group (n = 67)			
Phase II—Validation of screening phase by RT-qPCR				Phase III—Independent validation by RT-qPCR			
MicroRNA	*P *value	Log2 FC	Regulation	MicroRNA	*P *value	Log2 FC	Regulation
miR-144-5p	0.0297	− 1.90	Lower	miR-144-5p	0.0102	− 1.66	Lower
miR-7-1-3p	0.0138	− 1.62	Lower	miR-7-1-3p	0.0365	− 0.71	Lower
miR-126-3p	0.0083	− 1.51	Lower	miR-126-3p	0.0112	− 1.65	Lower
miR-146b-5p	0.0025	− 1.26	Lower	miR-146b-5p	0.0282	− 0.71	Lower
miR-338-3p	0.0116	− 1.41	Lower	miR-338-3p	0.0064	− 1.11	Lower
miR-3200-3p	0.0225	− 3.02	Lower	miR-3200-3p	0.0264	− 2.09	Lower
miR-330-3p	0.0206	− 1.55	Lower	miR-330-3p	0.0309	− 1.33	Lower
miR-21-5p	0.0076	− 1.53	Lower	miR-21-5p	0.0111	− 1.45	Lower
miR-7-5p	0.0120	− 2.20	Lower	miR-7-5p	0.0049	− 1.47	Lower
miR-660-5p	0.0090	− 1.21	Lower	miR-660-5p	0.0225	− 0.85	Lower
miR-148b-3p	0.0011	− 1.99	Lower				
miR-199b-5p	0.0355	− 1.33	Lower	**miRNAs detected only in the independent sample set**^**a**^			
				miR-502-5p	0.0339	− 1.36	Lower
				miR-27a-3p	0.0037	− 1.05	Lower
				miR-374b-5p	0.0285	− 1.01	Lower
				miR-140-5p	0.0144	− 1.15	Lower

HAMH (n = 13) compared to NAMH group (n = 13)				HAMH (n = 27) compared to NAMH group (n = 67)			
Phase II—Validation of Screening phase by RT-qPCR				Phase III—Independent validation by RT-qPCR			
MicroRNA	*P *value	Log2 FC	Regulation	MicroRNA	*P *value	Log2 FC	Regulation
miR-3125	0.0139	1.74	Higher	miR-100-5p	0.0006	− 2.67	Lower
miR-483-5p	0.0028	2.18	Higher	miR-3200-3p	0.0003	− 3.25	Lower
miR-100-5p	0.0001	− 3.87	Lower	miR-7-1-3p	0.0001	− 1.45	Lower
miR-3200-3p	0.0274	− 3.06	Lower	miR-18a-5p	0.0042	− 2.00	Lower
miR-7–1-3p	0.0279	− 1.49	Lower	miR-17-3p	0.0394	− 1.44	Lower
miR-18a-5p	0.0146	− 1.97	Lower	miR-133b	0.0091	− 1.18	Lower
miR-17-3p	0.0226	− 1.43	Lower	miR-221-3p	0.0046	− 1.34	Lower
miR-133b	0.0281	− 1.70	Lower	miR-18b-5p	0.0120	− 1.78	Lower
miR-654-3p	0.0412	− 1.27	Lower	miR-744-5p	0.0076	− 0.65	Lower
miR-221-3p	0.0194	− 1.68	Lower	miR-10a-5p	0.0310	− 0.78	Lower
miR-18b-5p	0.0063	− 2.02	Lower	miR-28-5p	0.0001	− 1.37	Lower
miR-744-5p	0.0201	− 0.60	Lower	miR-330-3p	0.0008	− 1.69	Lower
miR-10a-5p	0.0062	− 1.52	Lower	miR-660-5p	0.0115	− 1.02	Lower
miR-28-5p	0.0059	− 1.36	Lower	miR-183-5p	0.0019	− 1.41	Lower
miR-181a-2-3p	0.0003	− 1.92	Lower	miR-148b-3p	0.0008	− 1.53	Lower
miR-330-3p	0.0107	− 1.87	Lower				
miR-660-5p	0.0380	− 0.93	Lower	**miRNAs detected only in the independent sample set**^**a**^			
miR-183-5p	0.0155	− 1.47	Lower	miR-27a-3p	0.0001	− 1.70	Lower
miR-199b-5p	0.0050	− 2.93	Lower	miR-148a-3p	0.0090	− 1.49	Lower
miR-505-3p	0.0114	− 1.69	Lower	miR-7-5p	0.0035	− 1.57	Lower
miR-148b-3p	0.0125	− 1.39	Lower	miR-96-5p	0.0151	− 0.91	Lower
miR-199a-5p	0.0349	− 1.52	Lower	miR-500b-5p	0.0000	− 1.20	Lower
miR-23b-3p	0.0425	− 1.06	Lower	miR-144-5p	0.0108	− 1.80	Lower
				miR-140-5p	0.0270	− 1.11	Lower
				miR-338-3p	0.0002	− 1.55	Lower
				miR-182-5p	0.0002	− 3.16	Lower

Unpaired t-test was used to calculate the *P *value.

Benjamini–Hochberg correction for multiple testing was used to adjust the *P *value.

*P *values of < 0.05 was considered statistically significant.

Relative abundance level of 2^_ΔΔCt^ was used for RT-qPCR.

*LAMH* low anti-Müllerian hormone, *HAMH* high anti-Müllerian hormone, *NAMH* normal anti-Müllerian hormone, *FC* fold change.

^a^Additional miRNAs were only detected by RT-qPCR in the independent validation phase and were not previously detected by microarray in the screening phase.

Similarly, RT-qPCR showed the same direction of abundance changes as the microarray analysis for 23 miRNAs when comparing the samples from HAMH to NAMH (miR-3125, and miR-483-5p, miR-100-5p, miR-3200-3p, miR-7-1-3p, miR-18a-5p, miR-17-3p, miR-133b, miR-654-3p, miR-221-3p, miR-18b-5p, miR-744-5p, miR-10a-5p, miR-28-5p, miR-181a-2-3p, miR-330-3p, miR-660-5p, miR-183-5p, miR-199b-5p, miR-505-3p, miR-148b-3p, miR-199a-5p, and miR-23b-3p) (Table 3, phase II). Significant changes in abundance were confirmed for 2 miRNAs with higher abundance levels and 21 miRNAs with lower abundance levels (*P* < 0.05).

Further validation of the identified miRNAs was conducted by using a cohort of independent samples (phase III). Women with low (n = 27), high (n = 27), and normal (n = 67) levels of AMH level were included. As for the validation step, the RT-qPCR confirmed the direction of abundance changes and the significance of different abundances between LAMH and NAMH for 10 miRNAs (miR-144-5p, miR-7-1-3p, miR-126-3p, miR-146b-5p, miR-338-3p, miR-3200-3p, miR-330-3p, miR-21-5p, miR-7-5p, and miR-660-5p (*P* < 0.05) (Table 3). Additionally, out of the 23 identified miRNAs in the phase II between HAMH and NAMH groups, 15 miRNAs including miR-100-5p, miR-3200-3p, miR-7-1-3p, miR-18a-5p, miR-17-3p, miR-133b, miR-221-3p, miR-18b-5p, miR-744-5p, miR-10a-5p, miR-28-5p, miR-330-3p, miR-660-5p, miR-183-5p, and miR-148b-3p showed a significantly lower abundance level in HAMH compared to NAMH (*P* < 0.05) (Table 3).

Moreover, 4 miRNAs (miR-502-5p, miR-27a-3p, miR-374b-5p, and miR-140-5p) and 9 miRNAs (miR-27a-3p, miR-148a-3p, miR-7-5p, miR-96-5p, miR-500b-5, miR-144-5p, miR-140-5p, miR-338-3p, and miR-182-5p) were validated only in the independent sample set by RT-qPCR (phase III), when the number of samples have been increased (Table 3). By considering only the miRNAs which have been validated in phase III, we noticed that some miRNAs were deregulated in either one group (either LAMH or HAMH) or both groups (shared miRNAs) compared to NAMH. Specifically, 5 miRNAs were differentially abundant in only LAMH versus the NAMH group, 15 miRNAs in only HAMH versus NAMH group, and 9 miRNAs were shared in both groups (Fig. 1). When the two groups LAMH and HAMH were compared with one another, no significant difference in the abundance level was found, by using RT-qPCR either (data not shown).

### Correlation of the validated miRNAs with age and AMH

**Figure 2:**
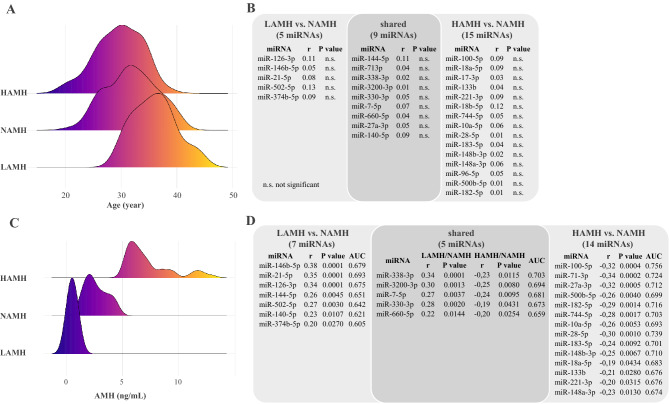
(**A**) Differences in the miRNA abundance level based on age, (**B**) correlation analysis of the differentially abundant miRNAs, which were validated exclusively in LAMH versus NAMH and HAMH versus NAMH with age, (**C**) Differences in the miRNA abundance level based on AMH (ng/ml), (**D**) Correlation analysis of the differentially abundant miRNAs, which were validated exclusively in LAMH versus NAMH and HAMH versus NAMH with AMH. Spearman's correlation; r, correlation coefficient; statistically significant if *P* value < 0.05. *LAMH* low anti-Müllerian hormone, *HAMH* high anti-Müllerian hormone, *NAMH* normal anti-Müllerian hormone, *AMH* anti-Müllerian hormone.

A woman's fertility gradually declines with age and this decline significantly correlates with the number and quality of her eggs. To exclude the age-related changes in miRNA abundance level, correlation analysis between the validated miRNA abundance levels (phase III, i.e., 29 miRNAs) (Fig. 1) and the age of the included women was performed. As shown in Fig. 2A, the miRNA abundance levels were shifted slightly towards older women with lower AMH levels (i.e., women with poor ovarian reserve) and were shifted slightly towards younger women with higher AMH levels. However, this slight shift in the abundance level of miRNAs was not significantly correlated to age, suggesting that the altered abundance level of miRNAs that we observed occurs regardless of age, as depicted in Fig. 2B. In contrast to age, the validated miRNA abundance levels (phase III, i.e., 29 miRNAs) in the LAMH, HAMH, and NAMH were mostly abundant in the range of low, high and normal AMH concentrations, respectively, as shown in Fig. 2C. To prove that the alteration in miRNA abundance level is due to the changes in AMH levels, a spearman's correlation was carried out. A significant correlation was observed for 26 out of 29 miRNAs in LAMH versus NAMH and HAMH versus NAMH (Fig. 2D). Of these correlated miRNAs, 7 miRNAs were correlated with AMH level in the LAMH versus NAMH group and 14 miRNAs in HAMH versus NAMH group. While 5 miRNAs were correlated with AMH in both tested groups i.e., LAMH versus NAMH and HAMH versus NAMH. These findings provide evidence that the miRNA abundance levels changed significantly in the groups, depending on the level of AMH.

Additionally, miR-144-5p and miR-140-5p that were previously shared in both groups (i.e., LAMH and HAMH, Fig. 1) were found to be correlated with AMH level in only LAMH versus NAMH. Similarly, miR-7-1-3p and miR-27a-3p were previously shared in both (i.e., LAMH and HAMH, Fig. 1), were found to be correlated with AMH level in only HAMH versus NAMH. Interestingly, the abundance level of miRNAs that were positively correlated with AMH in the LAMH versus NAMH is negatively correlated with the AMH in the HAMH versus NAMH (Fig. 2D, *P* < 0.05).

### Diagnostic accuracy of the validated miRNAs

The miRNAs that have been validated in phase III by RT-qPCR and correlated with AMH level (26 miRNAs, Fig. 2D, Supplementary Fig. 2) were tested for their suitability as biomarkers for assessing the ovarian reserve of a woman presenting at an infertility clinic. The receiver operating curve (ROC) analysis was performed, in which the miRNAs were tested for their predictive ability to detect women with low, normal, and high-ovarian reserve. All identified miRNAs that correlated with AMH have an AUC > 0.5. The best AUC value was observed for the miR-100-5p for the prediction of an increased AMH level with an AUC = 0.756. The calculated AUC values for the validated miRNAs correlated with AMH are shown in Fig. 2D.

## Discussion

In this study, the difference in miRNA abundance level was determined in women with normal, low, and high ovarian reserve by miRNA microarray and RT-qPCR analyses. Considering miRNAs with an adjusted *P* value < 0.05 exhibiting ≥ 2-fold change in abundance level, the abundance level of 34 and 98 miRNAs was significantly altered in the high (HAMH) and low LAMH groups, respectively, as compared to the normal NAMH group. The result was validated by RT-qPCR for 12 and 23 miRNAs in the LAMH and HAMH groups, respectively. Using an independent set of samples, 14 and 24 miRNAs were validated in LAMH and HAMH groups, respectively (Fig. 1). Despite that the mean age of the three groups differed significantly, statistical analysis indicated that no significant correlation exists between the abundance level of the validated miRNAs and the age of women in the three tested groups (Fig. 2B). On the other hand, the abundance level of the validated miRNAs was significantly correlated with the AMH level in the tested groups (Fig. 2D). Accordingly, we may argue that the changes in miRNA abundance level have resulted from differences in the AMH level rather than differences in the women's age. A statistically significant difference was observed between the three groups in terms of the mean age, PRL, LH, FSH, Basal E2, Testosterone, Androstenedione, DHEA-S, and AFC. Low levels of FSH and Basal E2 were found to correlate with improved ovarian response^17^. Higher levels of LH and testosterone contribute to early ovarian reserve failure^18,19^, while PRL could directly act on the ovary to suppress follicular development^20^. Additionally, higher level of PRL have been correlated with menstrual disorders because of its restraining effect on pulsatile Gonadotropin-releasing hormone (GnRH) secretion as well as inhibition of FSH and LH release^21^.

Our results concord with previous reports showing that many of our dysregulated miRNAs play a role in female reproduction and/or infertility-associated diseases of women and/or her male partner^3–11^. In more details, MiR-330-3p, miR-144-5p, and miR-221-3p showed lower abundance levels in the cumulus cells of women with polycystic ovary syndrome (PCOS)^22^, and miR-144-5p and miR-133b expression in the granulosa cells and was linked to female infertility^23^. In particular, miR-144-5p inhibits prostaglandin E2 (*PGE2*) secretion, which is an important regulator of ovulation and can therefore lead to fertility problems through changes in its synthesis^24^. Xiao et al*.* found a higher abundance level of miR-133b in meiosis I oocytes when the insulin-like growth factor-I (*IGF-1*) was overexpressed^25^. IGF-1 is involved in the development of the primordial follicles and the growth of the oocytes^25,26^. The abundance level of miR-140-5p was significantly decreased in the follicular fluid of women with PCOS as compared to controls^27^. Regulation of miR-140-5p by the Estrogen receptor α (*ERα*) was detected in women with breast cancer and in women with PCOS^27,28^. Similarly, miR-126-3p was also associated with PCOS^29^. The hypermethylation in the promoter site of the miR-126-3p gene was found in granulosa cells of patients with PCOS, which resulted in a reduction of the abundance level of miR-126-3p^29^. Another two miRNAs, namely miR-148a-3p and miR-28-5p were associated with endometriosis^30,31^. As for miR-148a-3p, He et al*.* identified it as a modulator for the Estrogen (E_2_)‐induced epithelial‐mesenchymal transition (*EMT*), which leads to endometriosis^31^. Besides, Liu et al*.* found that the reduced abundance level of miR-148a-3p leads to the higher abundance level of its target gene, the small nucleolar RNA host gene 4 (*SNHG4*), and thus has a direct influence on the ectopic growth of the endometrium outside the uterus^32^. As for miR-28-5p, Vanhie et al*.* concluded that miR-28-5p can be used as a non-invasive biomarker for endometriosis in infertile women^30^. Additionally, miR-28-5p was involved in the pathogenesis of PCOS via its target gene prokineticin 1 (*PROK1*) which plays a role in ovarian physiology, implantation of embryos in the endometrium, and success of pregnancies^33^.

MiR-21-5p was associated with the female reproductive system in many ways, besides its role in various types of cancer, such as breast^34^, ovarian^35^, and cervical cancer^36^. MiR-21-5p has been associated with diseases that affect female fertility including the development of endometriosis, PCOS, and primary ovarian insufficiency (POI). In-depth, miR-21-5p was found to promote angiogenesis associated with the development of endometriosis^37^. Park et al*.* observed an up-regulation of miR-21-5p in endometrial cells^38^, while Papari et al*.* found a reduced expression level of miR-21-5p in the plasma of women with endometriosis^39^. A physiological reduction in miR-21-5p was observed in human endometrial stromal cells (hESC) in preparation for pregnancy (decidualization)^40^. The abundance level of miR-21-5p was also investigated when the ovarian reserve was changed. Karakaya et al*.* found a higher abundance level of miR-21-5p in cumulus cells of women with low ovarian reserve (poor responder) presenting at an infertility clinic, while the abundance level of miR-21-3p was lower^41^, suggesting that elevated miR-21-5p abundance level in cumulus cells is not regulated at the pre-miR-21 level in women with low ovarian reserve. Very recently, a reduction in miR-21-5p abundance level was observed in the plasma of infertile women with abnormal AMH levels^42^. Overall, it became clear that the miR-21-5p is changed in many processes that are related to the ovarian reserve. It can be assumed that miR-21-5p is a key regulator of female fertility.

MiR-100-5p has recently been identified as a potential biomarker for various female reproduction disorders, in ectopic pregnancies^43^, endometriosis^44^, recurrent implantation failure^45^, and decreased ovarian reserve^46^. A lower expression level of miR-100-5p was found in women with ectopic pregnancies compared to that in normal pregnancies^43^. Similarly, a reduction in miR-100-5p expression level was observed in serum and plasma of women with unsuccessful embryo transfer compared to successful embryo transfer^45^, suggesting that miR-100-5p, may serve as a potential biomarker for recurrent miscarriage and/or recurrent implantation failure^38^. Woo et al*.* also observed a reduction in miR-100-5p expression level in granulosa cells of women with diminished ovarian response^46^ and reported that miR-100-5p targets fibroblast growth factor receptor 3 (*FGFR3*), insulin-like growth factor 1 receptor (*IGF1R*), and cyclin E and cyclin-dependent kinase (CDK), which play a role in the proliferation and steroidogenesis of granulosa cells. Therefore, Woo et al*.* concluded that miR-100-5p limits the proliferation of granulosa cells by binding to these target genes. In agreement with our finding, miR-100-5p decreased in abundance in women with lower and higher ovarian reserve compared to normal suggesting that this miRNA can be used as a biomarker for ovarian reserve in women undergoing infertility treatment. Based on these studies, our finding of an altered abundance of miRNAs (especially MiR-21-5p and miR-100-5p) in women with reduced or increased ovarian reserve suggests that they may be involved in the pathogenesis of the condition and lay the ground for the development of novel diagnostic approaches for women manifesting diminished ovarian reserve and subsequent fertility complications.

Various parameters and biomedical markers have been proposed to detect the ovarian reserve including age, FSH, Estradiol, Inhibin, AMH, and AFC^1,47^. Of these markers, AFC and AMH levels have been considered good predictors of the ovarian reserve during ART compared with other traditional measures^1,47–50^. AMH level was observed to be highly correlated with the AFC, age, and basal FSH level^1,49^. Therefore, AMH has been labelled as an adequate predictor of the ovarian reserve before IVF/ICSI treatment, in both, high and poor ovarian responders^1,47–50^. Although the AMH level is highly correlated with the AFC, in clinical practice there is a discrepancy between the AMH level and the AFC in about 18–32% of women presenting at an infertility clinic^48,51^. In our study, due to the controversial discussion over the decades between the AMH and AFC, we opted to classify our included women based on the AMH level and because all included women in our study have been tested for AMH, but not for AFC. Furthermore, AMH was correlated with LH, FSH, testosterone, and androstenedione and this is likely to be attributed to the interaction of other hormones during the menstrual cycle. The age (negatively correlated) and the AFC (positively correlated) were more significantly correlated with the AMH than to other markers and this is in agreement with the existing literature^1,47–51^. Age is the single biggest factor affecting a woman's chance to achieve pregnancy, and therefore age cannot be ignored in infertility research. It has been shown that a relevant portion of human miRNA changes depending on age and sex^52^. In our study, age was highly correlated with the AMH level, but not with the miRNA abundance level of the validated miRNAs, and thus provide evidence that the alteration in miRNA abundance level was not linked to age, and most probably linked to the changes in AMH level. The AMH level was positively correlated with the abundance level of miRNAs in LAMH versus NAMH group and negatively correlated with the abundance level of miRNAs in HAMH versus NAMH group, indicating that when the AMH level decreases, the abundance level of the identified miRNAs decreases and when the AMH level increases, the miRNA abundance level also decreases. Accordingly, an abnormal AMH level always leads to a reduction in the abundance level of miRNAs. This could explain the observed non-significant correlation and non-significant alteration in the miRNA abundance level in the screening and validation phases when the two abnormal groups (i.e., LAMH versus HAMH) were compared to one another. These findings suggest that miRNA abundance level is always reduced in case of an abnormal AMH level and probably in the cases with manifestations associated with diminished ovarian reserve. Based on these findings it is, however, premature to draw such a conclusion, as the number of included subjects in each group is too low.

One of the primary goals of this study was to find a diagnostic biomarker that can assess a woman's ovarian reserve and thus her fertility and to supplement or replace previously used biomarkers. The altered miRNAs that are correlated with AMH were therefore subjected to a ROC analysis, which allows a statement to be made about the quality of the miRNA as a biomarker. For this purpose, the AUC values ​​were determined and found greater than 0.5 in all validated miRNAs correlated with AMH in both comparison groups LAMH versus NAMH and HAMH versus NAMH. This means that the miRNAs identified in the LAMH versus NAMH group can predict a low AMH level and thus a low ovarian reserve. MiRNAs identified as biomarkers in HAMH versus NAMH can predict a high AMH level and thus a high ovarian reserve. MiRNAs identified in both groups can predict abnormal AMH levels or abnormal ovarian reserve. The miRNAs, which can predict either low or high ovarian reserve, would tend to be more useful as biomarkers based on the information about the current state of the ovarian reserve. The best AUC value was determined for the miR-100-5p. It indicates with a probability of 76% that the ovarian reserve is higher than normal.

In summary, the alteration in miRNA abundance level is in part associated with female reproduction and with diseases that affect female fertility status, and other miRNAs were newly identified in this area, suggesting that some miRNAs are involved in the maintenance of female fertility and changes of some other miRNAs might adversely and /or negatively affect female fertility. Altered abundance levels of miRNAs, particularly miR-100-5p, can provide new insights into the underlying mechanisms of female fertility and thus improve the diagnosis and treatment of infertility. The identified and validated miRNAs in our study should be further validated in a larger number of samples to confirm their predictive ability as biomarkers that could complement or possibly replace the previous markers as genetic biomarkers.

## Methods

### Study population and sample collection

Blood samples were collected from a total of 159 consecutive women undergoing infertility treatment with IVF-ICSI at Saarland University School of Medicine IVF Center (Homburg/Saar) between November 2016 and May 2020. The mean age was 32 ± 4.8 years (range 20–44 years). At enrollment, ultrasonography was conducted on the second day of the menstrual cycle to evaluate the anatomical characteristics of the female reproductive system and determine the antral follicular count (AFC). Peripheral blood samples were then collected from each woman into serum tubes and PAXgene blood tubes (Becton–Dickinson, Heidelberg, Germany) on the third day of the menstrual cycle. The serum was immediately prepared by centrifugation at 1800*g* for 15 min and used to determine the level of Free Thyroxine 4 (FT4), Thyroid-Stimulating Hormone, Prolactin (PRL), Luteinizing Hormone (LH), Follicle Stimulating Hormone (FSH), Estradiol (E2), Testosterone, Androstenedione, Dehydroepiandrosterone Sulfate (DHEA-S), and anti-Müllerian hormone (AMH). All PAXgene blood tubes were stored at room temperature for at least 24 h to ensure complete lysis of the blood cells, then stored at − 20 °C for several days and finally transferred to − 80 °C for long-term storage until RNA including miRNA isolation. This study was approved by the Saarland University Institutional Review Board committee (Ärztekammer des Saarlandes Nr. 160/15) and informed consent was obtained from each participant and the study complies with the Declaration of Helsinki.

The determination of miRNA abundance levels was performed in three successive phases as indicated in Fig. 1. In phase I (screening phase), samples were randomly grouped based on their AMH level into low AMH level (LAMH, n = 12), normal AMH level (NAMH, n = 13), and high AMH level (HAMH, n = 13).

These samples were used to identify the differential miRNA abundance level by applying a miRNA microarray. In phase II (validation of the screening phase), the initially identified miRNAs (phase I) were evaluated, by RT-qPCR assay. In phase III (Validation of the independent cohort), the selected miRNAs in phase II were additionally validated by RT-qPCR in an independent set of samples from 27, 67, and 27 women from the low, normal, and high level of AMH, respectively.

### Isolation of total RNA, including miRNAs

Total RNA including miRNAs was isolated from blood samples using PAXgene Blood miRNA Kit on the QIAcube robot (Qiagen, Hilden, Germany) following the manufacturer's recommendations. DNase I treatment (Qiagen, Hilden, Germany) was carried out during the isolation to eliminate any genomic DNA contamination as previously described^9^. The total RNA concentration was measured using the NanoDrop ND-2000 spectrophotometer (Thermo Fisher Scientific, Massachusetts, United States). RNA purity was assessed by determining the OD 260/280 and the OD 260/230 ratios. The quality of total RNA was assessed using the Agilent Bioanalyser 2100 Eukaryote Total RNA Nano Series II (Agilent Technologies, California, United States).

### MiRNA microarray

MiRNA expression profiles in LAMH, NAMH, and HAMH were determined by hybridization to the Sureprint G3 Human v21 miRNA microarray chips, 8 × 60 K (release 21.0), each containing 2549 human miRNAs (Agilent Technologies). Hybridizations were carried out following the manufacturer's recommendations. In brief, 120 ng RNA from each sample was processed using the miRNA Complete Labeling and Hybridization Kit (Agilent Technologies) to generate fluorescence-labeled miRNA. The microarrays were loaded and incubated at 55 °C for 20 h with rotation. After several washing steps, microarrays were scanned with the Agilent Microarray Scanner at 3 microns in double path mode. Data was acquired using Agilent AGW Feature Extraction software version 10.10.11 (Agilent Technologies).

### Reverse transcription and quantitative real-time PCR (RT-qPCR) of miRNA

The abundance level of circulating miRNAs was quantified by RT-qPCR using the Biomark HD System (Fluidigm Corporation, California, United States) and the TaqMan microRNA Assays (Thermo Fisher Scientific) according to the as previously described^53^. Briefly, complementary DNA (cDNA) was generated in 8 µL reactions by reverse transcription of 350 ng total RNA using the TaqMan MicroRNA Reverse Transcription Kit and RT Primers Pool (10×) (Thermo Fisher Scientific). Following reverse transcription, 2.5 μL of the generated cDNA was preamplified by mixing 12.5 µL of TaqMan PreAmp Master Mix (2×) and 3.75 μL of PreAmp Primers Pool (10×) (Thermo Fisher Scientific) in 25 µL reaction volume. Following the preamplification of the cDNA, RT-qPCR was carried out with 96.96 Dynamic Array IFC for Gene Expression arrays (Fluidigm Corporation) as indicated in Fluidigm's protocol (PN 68000130 E1). Briefly, every 10× Assays contained 3 µL TaqMan Primer Assay (20×) (a mixture of forward and reverse primers, and probe) (Thermo Fisher Scientific), and 3 µL Assay Loading Reagent (2×) (Fluidigm, PN 85000736). Sample Pre-Mix was prepared by combining 3 µL TaqMan Universal PCR Master Mix, no AmpErase™ UNG (2×) (Thermo Fisher Scientific), 0.3 µL GE Sample Loading Reagent (20×) (Fluidigm, PN 85000735), and 2.7 µL pre-amplified cDNA for each sample. Finally, 5 µL of each **A**ssay and **S**ample **M**ix were transferred into the appropriate inlets according to the Fluidigm's recommendation. After loading, the array was placed in the Biomark HD instrument for quantification and detection using GT 96 × 96 Standard v1 PCR thermal protocol. The data were analyzed with Real-Time PCR Analysis Software (Fluidigm Corporation) according to Fluidigm's recommendation. Negative control samples (H_2_O) were included in the reverse transcription, preamplification, and amplification steps and were finally defined as those with Ct values ≥ 35 or undetermined.

### Statistical analysis

Microarray images were scanned using the Feature Extraction Software (Agilent Technologies) and the extraction of data was carried out using GeneSpring GX software (version 14.9.1, Agilent Technologies). Microarray measurements were normalized using quantile normalization and the differential abundance levels of miRNAs were identified for each sample. An unpaired two-sample t test with Benjamini–Hochberg correction for multiple testing was applied, and adjusted *P *value of < 0.05 was considered statistically significant. Fold change for the LAMH and HAMH groups was obtained with respect to the NAMH group. Consequently, the lower and higher abundant miRNAs with a fold change > 2 were considered for further studies. The diagnostic value for each validated miRNA was evaluated by receiver operating characteristic (ROC) curves analysis and subsequently the area under the ROC curve (AUC) was computed to assess the potential use of miRNA(s) as a biomarker. The relative quantitative method of 2^−ΔΔCt^ was used to measure the dynamic change of selected validated miRNAs using RNU6B small nuclear RNA (snRNA) as an endogenous reference miRNA as previously validated for this type of sample^53–61^. The correlation analysis was carried out by the Spearman correlation coefficient and the differences in clinical characteristics among the three tested groups i.e., LAMH, HAMH, and NAMH were analyzed by analysis of variance (ANOVA) and data was presented as mean ± standard deviation with a *P *value of < 0.05 was considered statistically significant.

### Ethics approval

Institutional Review Board approval/Ethikvotum Ärztekammer des Saarlandes: Ethical vote No. 160/15.

## Supplementary Information

Supplementary Information.
