# Supplementary material for: Characterization of micro-RNA in women with different ovarian reserve
Source: Sci Rep. 2021 Jun 25;11:13351. doi: 10.1038/s41598-021-92901-w (PMC8233349; doi:10.1038/s41598-021-92901-w)

**Characterization of Micro-RNA in women with different ovarian reserve**

Masood Abu-Halima, Ph.D., ^1^ Lea-Simone Becker M.Sc., ^1^, Basim M. Ayesh, Ph.D., ^2^ Simona-Lucia Baus, Ph.D., ^3^ Amer Hamza, Ph.D., ^3,4^ Ulrike Fischer, Prof. Dr., ^1^ Mohamad Hammadeh, Prof. Dr.,^3^ Andreas Keller, Prof. Dr., ^5^ and Eckart Meese, Prof. Dr., ^1^

^1^ Institute of Human Genetics, Saarland University, 66421 Homburg/Saar, Germany.

^2^ Department of Laboratory Medical Sciences, Alaqsa University, Gaza, Palestine.

^3^ Department of Obstetrics and Gynecology, Saarland University, 66421 Homburg/Saar, Germany.

^4^ Kantonspital Baden, Im Ergel 1, 5400 Baden, Switzerland

^5^ Chair for Clinical Bioinformatics, Saarland University, 66123 Saarbruecken, Germany.

**Corresponding author**

Masood Abu-Halima, Ph.D.

Institute of Human Genetics

Saarland University

66421 Homburg/Saar

Germany

masood@daad‑alumni.de

**Supplementary Tables**

**Supplementary Table 1:** Correlation of AMH with demographic, hormonal, and parametric characteristics of women presenting at an infertility clinic

| **Spearman's Correlation** | | |
| --- | --- | --- |
| **Variables** | **r** | **P value** |
| Age | -0.491 | 0.001 |
| AFC | 0.694 | 0.001 |
| FSH | -0.452 | 0.003 |
| Testosterone | 0.374 | 0.018 |
| LH | 0.339 | 0.033 |
| Androstenedione | 0.314 | 0.048 |
| TSH | 0.285 | 0.075 |
| Estradiol | -0.132 | 0.415 |
| DHEA-S | 0.074 | 0.650 |
| PRL | 0.033 | 0.838 |
| FT4 | -0.020 | 0.903 |

- n = 159.
- Spearman’s correlation. r, correlation coefficient.
- Statistically significant if p-value <0.05.
- AMH, anti-Müllerian hormone; AFC, Antral Follicle Count; FSH, Follicle Stimulating Hormone; LH, Luteinizing Hormone; TSH, Thyroid Stimulating Hormone; Basal E2, Basal Estradiol; DHEA-S, Dehydroepiandrosterone Sulfate; PRL, Prolactin; FT4, Free Thyroxine

**Supplementary Figure 1:** List of miRNAs used for the RT-qPCR validation in the LAMH *versus* NAMH and HAMH *versus* NAMH groups. LAMH, Low anti-Müllerian hormone; HAMH, High anti-Müllerian hormone; NAMH, Normal anti-Müllerian hormone.


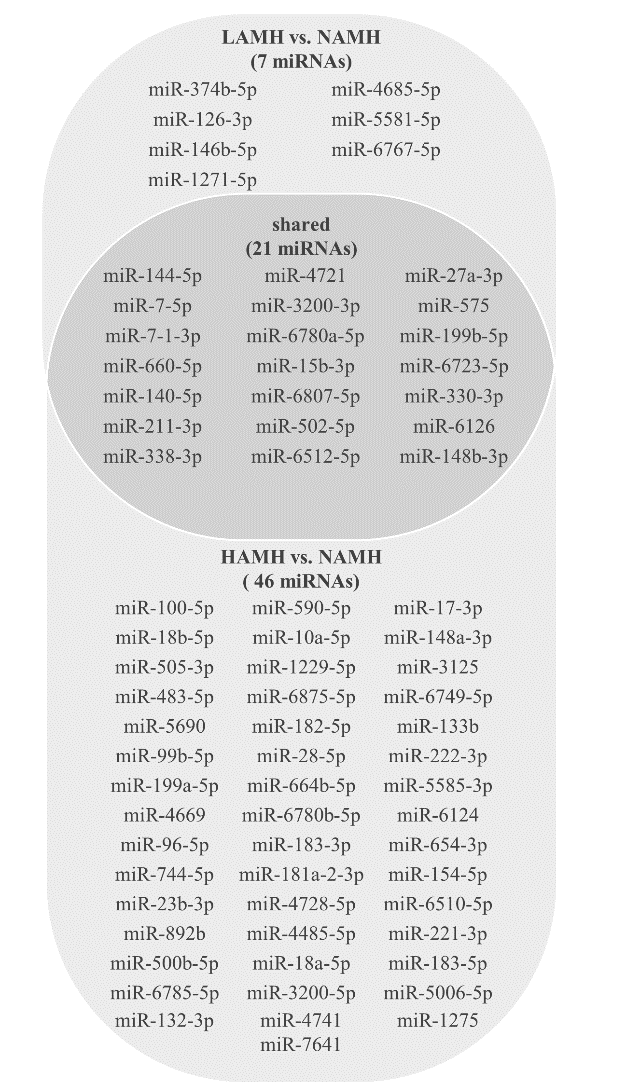


**Supplementary Figure 2:** The receiver operating curve (ROC) analysis for the for the validated miRNAs in phase III by RT-qPCR and correlated with AMH level HAMH vs. NAMH (14 miRNAs), LAMH vs. NAMH (7 miRNAs), and Shared miRNAs (5 miRNAs)


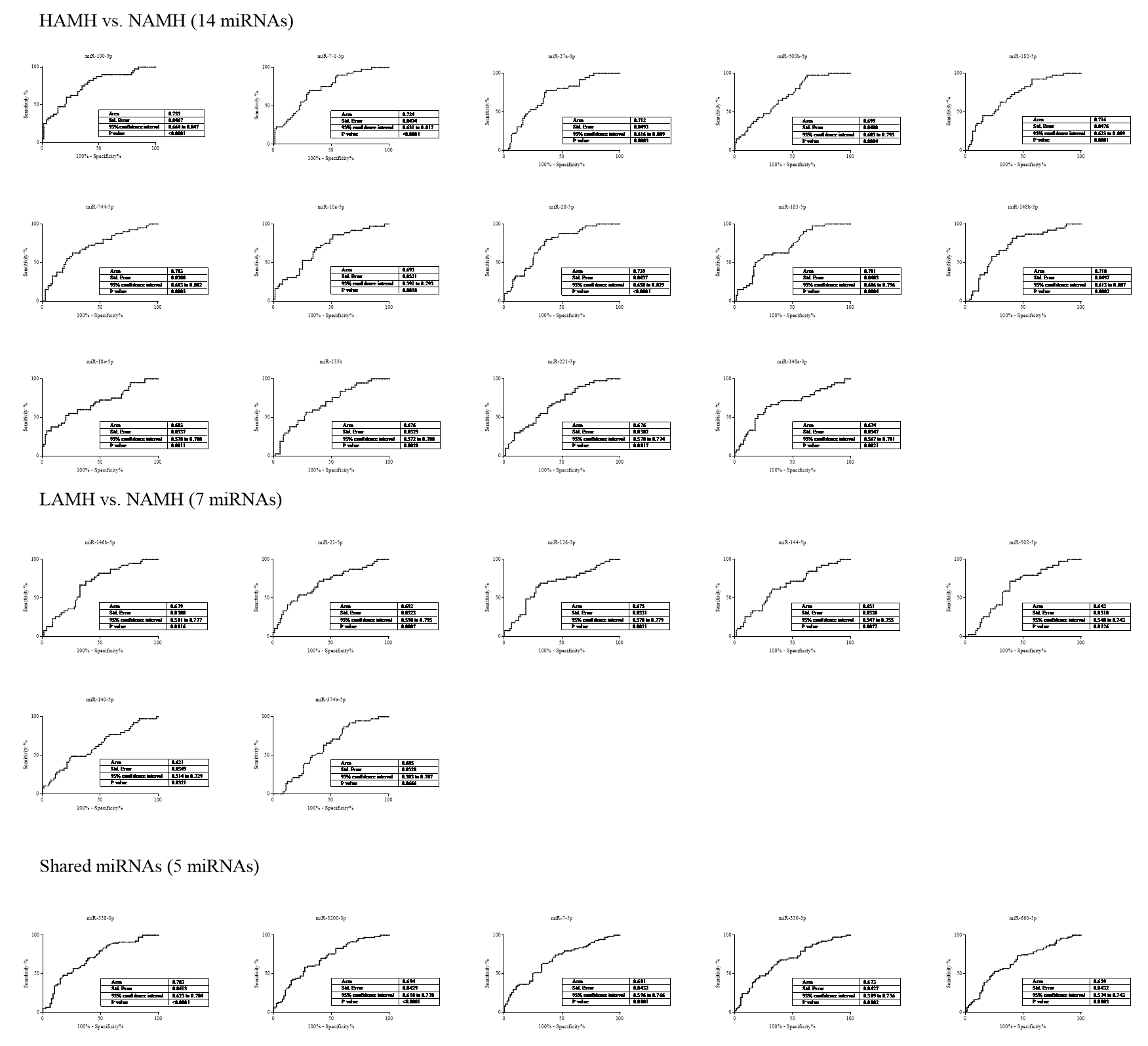

Supplement: Supplementary file 1 — Supplementary Information. [file 41598_2021_92901_MOESM1_ESM.docx]
